# A Rare Case of Poorly Differentiated Lower Esophageal Cancer with Neuroendocrine Differentiation and α-Fetoprotein Production

**DOI:** 10.70352/scrj.cr.25-0619

**Published:** 2026-02-20

**Authors:** Mamiko Takii, Masashi Takemura, Tsutomu Oshima, Masanori Yamada, Daiki Inazu, Hironari Miyamoto, Hiroki Yamaguchi

**Affiliations:** Department of Gastrointestinal Surgery, Minami Osaka Hospital, Osaka, Osaka, Japan

**Keywords:** esophageal cancer, neuroendocrine carcinoma, α-fetoprotein

## Abstract

**INTRODUCTION:**

Neuroendocrine differentiation and α-fetoprotein production (AFP) are 2 rarely reported features in poorly differentiated gastric carcinomas. We report an extremely rare case of esophageal carcinoma (EC) featuring both histologic types.

**CASE PRESENTATION:**

A 67-year-old man was referred to our hospital with dysphagia and weight loss. Upper gastrointestinal endoscopy revealed a type 3 tumor in the lower esophagus, 32–40 cm from the incisors, with luminal stenosis. The endoscopic biopsy specimen showed pathologic features of poorly differentiated carcinoma, and immunostaining revealed findings consistent with neuroendocrine carcinoma (NEC). CT showed no evidence of distant metastasis, although signs of metastatic lymph nodes were observed in the mediastinum and lesser curvature of the stomach. The patient declined chemotherapy and underwent thoracoscopic esophagectomy, which revealed advanced lymph node metastasis, particularly involving the left membranous trachea and pancreas. The resected tumor measured 90 × 80 mm and exhibited a type 3 pattern; the tumor center was located in the lower esophagus. Histologic examination of the resected specimen revealed Barrett’s esophagus (BE), with no continuity observed between the tumor and BE. The final diagnosis was poorly differentiated carcinoma with neuroendocrine and enteroblastic components. Despite extensive postoperative progression of lymph node metastasis revealed by CT, the patient achieved a complete response following the administration of chemotherapy and immune checkpoint inhibitors.

**CONCLUSIONS:**

Accumulating evidence suggests that the coexistence of hepatoid adenocarcinoma and neuroendocrine differentiation is associated with poor prognosis in EC, and further studies are warranted to develop specific treatment criteria for adjuvant therapy in patients with EC harboring these features.

## Abbreviations

5-FU
: 5-fluorouracil
AFP
: α-fetoprotein
BE
: Barrett's esophagus
CDDP
: cisplatin
CPS
: combined positive score
CR
: complete response
EC
: esophageal carcinoma
ENT
: enteroblastic
INSM1
: insulinoma-associated protein 1
NEC
: neuroendocrine carcinoma
SCC
: squamous cell carcinoma

## INTRODUCTION

Esophageal NEC is very rare, accounting for approximately 0.6% of all EC, according to the 2016 registry on esophageal cancer collated by the Japan Esophageal Society.^1)^ Conversely, AFP production is a relatively common feature of hepatocellular carcinoma and yolk sac tumors and has been reported in other cancers, including gastric, lung, pancreatic, and colorectal cancers.^2)^ AFP-producing esophageal cancer is an extremely rare, with few cases reported to date. Herein, we report a case of AFP-producing hepatoid EC with neuroendocrine differentiation.

## CASE PRESENTATION

**Fig. 1:**
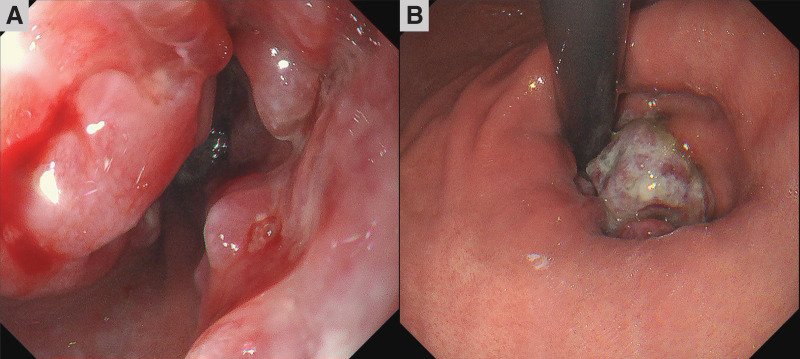
Upper gastrointestinal endoscopy findings. Upper gastrointestinal endoscopy revealed a type 3 tumor with circumferential irregular margins in the lower esophagus, extending into the esophagogastric junction (**A**, **B**).

**Fig. 2:**
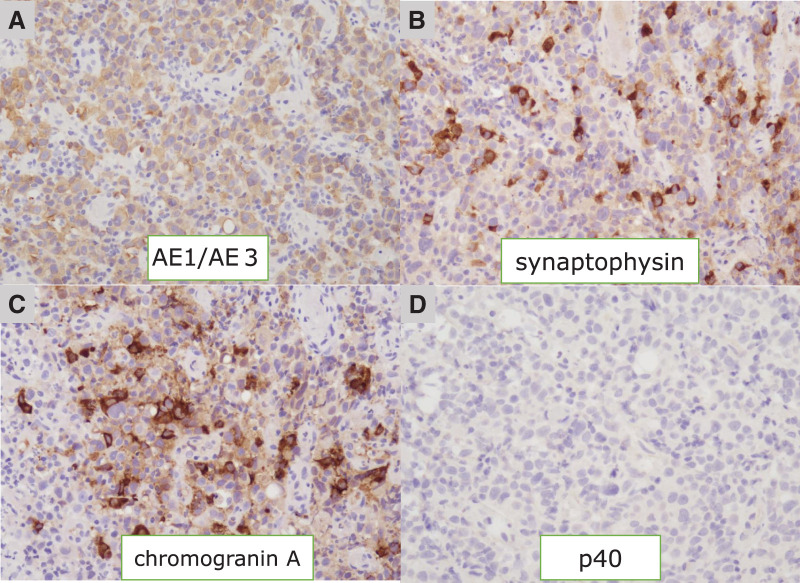
Histological findings of the biopsy specimen of the tumor. The tumor was a poorly differentiated carcinoma diffusely positive for anti–pan cytokeratin (AE1 + AE3) (**A**), partially positive for synaptophysin (**B**) and chromogranin A (**C**), and negative for p40 (**D**), suggesting NEC. NEC, neuroendocrine carcinoma

A 67-year-old man presented to our hospital with the chief complaints of dysphagia and weight loss. The patient had a history of myocardial infarction, asthma, and atopic dermatitis. The serum tumor marker levels were as follows: squamous cell carcinoma antigen, 14.3 ng/mL (normal range, <1.5 ng/mL); carcinoembryonic antigen, 14.3 ng/mL (normal range, <5.0 ng/mL); and cytokeratin 19 fragment, 9.8 ng/mL (normal range, <3.5 ng/mL). Upper gastrointestinal endoscopy revealed a type 3 tumor with circumferential irregular margins in the lower esophagus, extending into the esophagogastric junction (**Fig. 1A** and **1B**). The lumen was stenotic because of the tumor. Histopathologic evaluation of the endoscopic biopsy specimen revealed proliferation of atypical cells exhibiting mildly reduced cellular cohesion and an increased nuclear-to-cytoplasmic ratio. By immunohistochemistry, the tumor was a poorly differentiated carcinoma diffusely positive for anti–pan cytokeratin (AE1 + AE3), partially positive for synaptophysin and chromogranin A, and negative for p40, suggesting NEC (**Fig. 2A**–**2D**). The pathologist diagnosed NEC; however, an additional comment noted that the growth of atypical cells with somewhat weak cohesion was observed, which is atypical for a classic NEC.

**Fig. 3:**
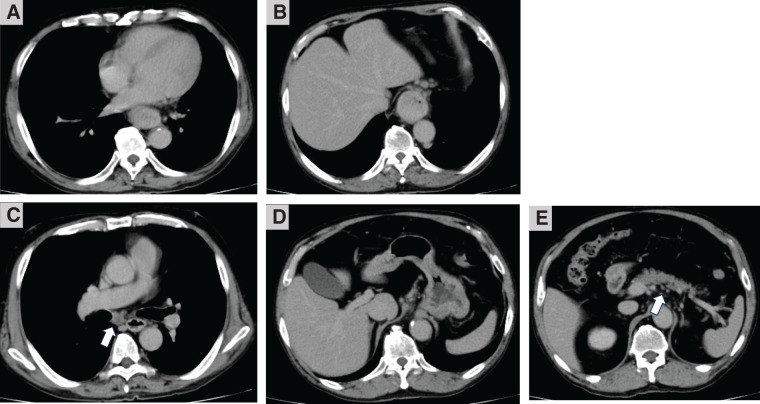
CT findings before esophagectomy. CT revealed wall thickening from the lower thoracic (**A**) to the abdominal esophagus (**B**), as well as enlarged bifurcational lymph nodes (**C**), lesser curvature of the stomach (**D**), and suprapancreatic lymph nodes (**E**). White arrows: enlarged lymph nodes.

CT revealed wall thickening extending from the lower thoracic to the abdominal esophagus (**Fig. 3A** and **3B**), no evidence of distant metastasis, and enlarged lymph nodes in the mediastinum and lesser curvature of the stomach (**Fig. 3C** and **3D**). This patient was classified as cStage III by the Japanese Classification of Esophageal Cancer, 11th edition.

The patient declined chemotherapy and underwent thoracoscopic esophagectomy due to the progression of stenotic symptoms. During surgery, extensive lymph node metastases were in contact with the left tracheoesophageal groove and the pancreas; therefore, noncurative resection was performed (**Fig. 3C** and **3E**, white arrows). Thoracoscopic surgery was performed with R2 radical esophagectomy. The thoracic esophagus was transected, leaving a sufficient length. The reconstruction route was via the retrosternal space. Gastric tube reconstruction was done with cervical esophagogastric anastomosis.

**Fig. 4:**
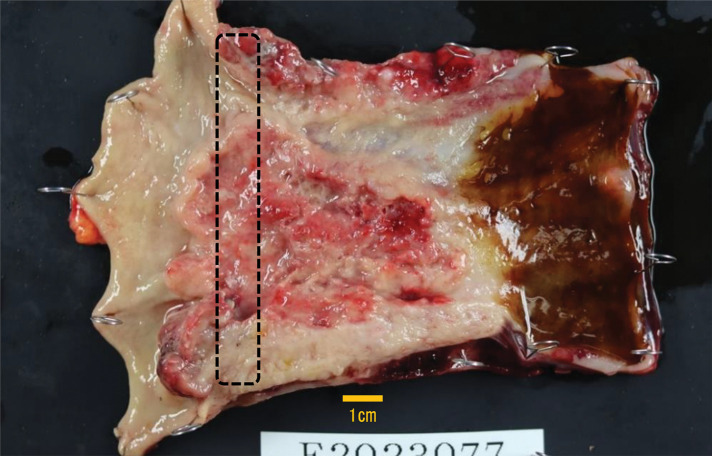
Resected specimen findings. The resected specimen of the esophagus, which was primarily located in the lower esophagus, extended into the esophagogastric junction. BE is in the dotted frame. BE, Barrett's esophagus

**Fig. 5:**
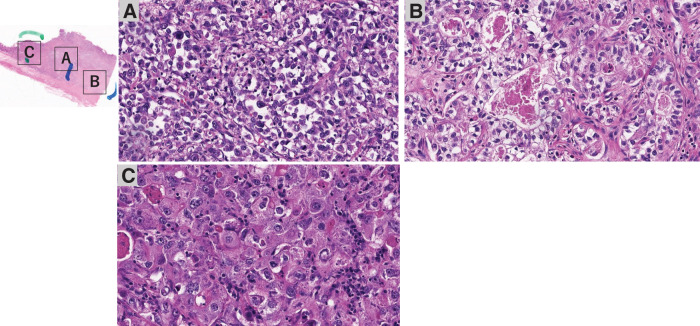
Results of surgical specimen pathology. Histopathologic examination revealed that the tumor comprised 3 different histological types of carcinoma: atypical cells exhibiting mildly reduced cellular cohesion and increased nuclear/cytoplasmic ratio (**A**); cells with a clear cytoplasm forming glandular structures, suggesting a fetal gastrointestinal-like adenocarcinoma (**B**); and cells with an abundantly eosinophilic cytoplasm, suggesting a hepatoid adenocarcinoma (**C**).

**Fig. 6:**
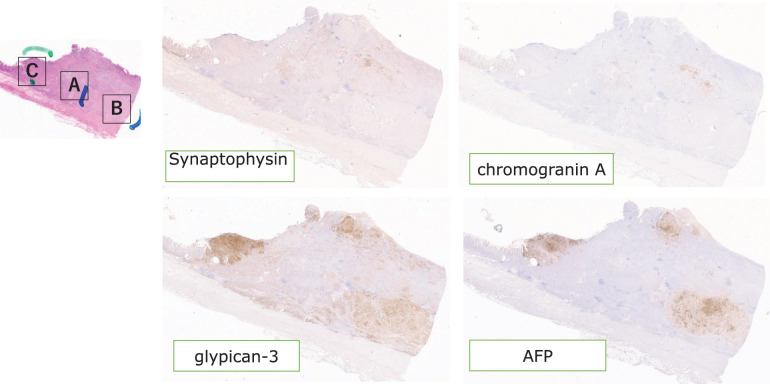
Results of surgical specimen immunohistochemical pathology. Immunohistochemical analysis revealed that area A was positive for synaptophysin and chromogranin A, whereas positivity for glypican-3 and AFP was mainly observed in areas B and C. Area A also had small intermingling areas of cells positive for glypican-3 and AFP. These immunohistochemical findings led to the definitive diagnosis of poorly differentiated carcinoma with 3 histological types: neuroendocrine differentiation, fetal gastrointestinal-like adenocarcinoma, and hepatoid adenocarcinoma. AFP, α-fetoprotein production

**Fig. 7:**
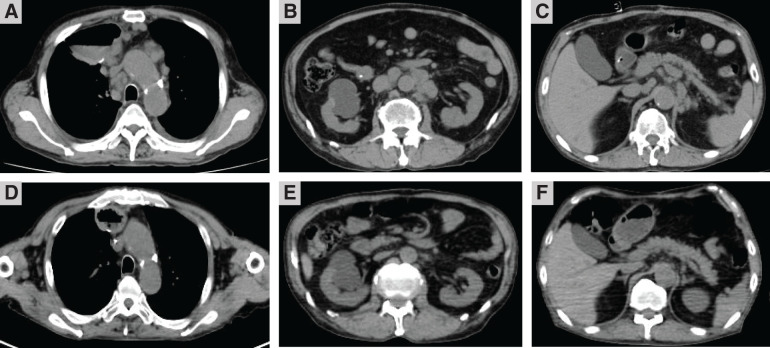
CT findings. CT shows extensive lymph node metastases involving the mediastinum, abdomen, and para-aortic area before immunochemotherapy (**A**–**C**), and CR after immunochemotherapy (**D**–**F**). CR, complete response

**Fig. 8:**
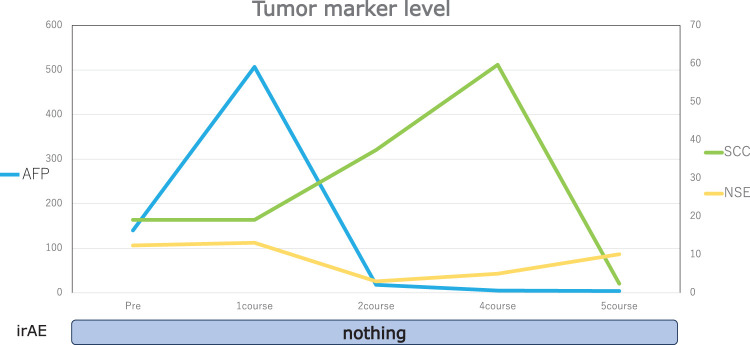
Tumor marker levels. All tumor markers, including AFP, NSE, and SCC antigen levels, declined below the normal range. AFP, α-fetoprotein production; irAE, immune-related adverse event; NSE, neuron-specific enolase; SCC, squamous cell carcinoma

On gross examination, the resected specimen, which was primarily located in the lower esophagus and extended into the esophagogastric junction, showed intramural metastasis in the upper thoracic esophagus (**Fig. 4**). Histopathologic examination of the resected tumor revealed the proliferation of atypical cells exhibiting mildly reduced cellular cohesion and an increased nuclear-to-cytoplasmic ratio, similar to the endoscopic biopsy findings, in 95% of the resected tumor (**Fig. 5A**). However, the remaining tumor included 2 distinct features: an area of proliferating atypical cells with a clear cytoplasm that formed glandular structures, suggesting a fetal gastrointestinal-like adenocarcinoma, and another area of solid, proliferating atypical cells with an abundantly eosinophilic cytoplasm, suggesting a hepatoid adenocarcinoma (**Fig. 5B** and **5C**). Immunohistochemical analysis revealed that the atypical cells exhibiting mildly reduced cellular cohesion were positive for synaptophysin and chromogranin A (**Fig. 6**), whereas positivity for glypican-3 and AFP was observed in clusters of atypical cells with a clear cytoplasm and in those with an abundantly eosinophilic cytoplasm. Atypical cells with the poorly differentiated morphology observed in the center of the specimen included small intermingling areas of cells positive for glypican-3 and AFP. These immunohistochemical findings led to the definitive diagnosis of poorly differentiated carcinoma with 3 histological types: neuroendocrine differentiation, fetal gastrointestinal-like adenocarcinoma, and hepatoid adenocarcinoma. Despite the presence of BE observed within the excised tumor specimen (**Fig. 4**, dotted frame), continuity between the tumor and BE could not be confirmed, and carcinoma components were not found in any areas of BE. A large number of the dissected lymph nodes extending from the mediastinum to the abdomen included metastatic tumor cells. Anti–programmed cell death ligand 1 testing revealed a CPS of >10, and the tumor cells were negative for microsatellite instability. CT performed 2 months after surgery detected extensive lymph node metastases through the cervical, mediastinal, and para-aortic regions (**Fig. 7A**–**7C**). The AFP level was high (140 ng/mL). The patient was initiated on combined chemotherapy with 5-FU (800 mg/m^2^, 82.5% dose), CDDP (80 mg/m^2^, 80% dose), and pembrolizumab (200 mg/body), achieving CR after 5 courses (**Fig. 7D**–**7F**). Moreover, all tumor markers, including AFP, neuron-specific enolase, and squamous cell carcinoma antigen levels, declined below the normal range (**Fig. 8**). At the last follow-up evaluation 18 months after surgery, the patient remained in CR with pembrolizumab therapy.

## DISCUSSION

According to the Japan Esophageal Society, squamous cell carcinoma is the most frequent histologic type in patients with EC, accounting for 87.5% of all cases, followed by adenocarcinoma, accounting for 7.2% of all cases, whereas other histologic types are uncommon.^1)^ Among these, esophageal NEC is histopathologically determined based on the rate of Ki-67 positivity and the presence of mitotic figures. According to the 2019 World Health Organization classification for digestive system tumors, neuroendocrine neoplasms are categorized as well-differentiated neuroendocrine tumors, poorly differentiated NECs, and mixed neuroendocrine–non-neuroendocrine neoplasms.^3)^ NEC is a very rare pathological type of EC, accounting for approximately 0.6% of all cases.^1)^ Among poorly differentiated neuroendocrine tumors, those with a Ki-67 index exceeding 20% are defined as large- or small-cell–type NECs without the G3 designation.^4)^ The diagnosis of NECs is based on immunostaining for synaptophysin, chromogranin A, and INSM1, given the high sensitivity of synaptophysin for NEC.^5,6)^ AFP-producing adenocarcinomas can be classified into fetal gastrointestinal-like, hepatoid, and yolk sac tumor–like adenocarcinomas.^7,8)^ Fetal gastrointestinal-like adenocarcinomas consist of columnar cells with clear cytoplasm, resembling fetal gastrointestinal epithelial tissue. Hepatoid adenocarcinomas are characterized by a solid growth pattern of cells with rich, pale eosinophilic cytoplasm.^9)^ AFP-producing carcinomas are specifically immunoreactive to AFP and glypican-3. Normally expressed in fetal hepatocytes, glypican-3 is not found in adult hepatocytes; however, various tumors, including hepatocellular carcinoma, hepatoblastoma, melanoma, testicular germ cell tumors, and Wilms tumors, have been reported to express glypican-3.^9)^ Compared to AFP-producing tumors arising in BE, those arising from the esophageal epithelium are more rarely reported. In the present case, the tumor extended from the lower esophagus across the esophagogastric junction into the stomach. The specimen included some BE lesions, but no evidence of carcinoma was found within BE. Thus, whether the tumor arose from a background of squamous epithelium or BE could not be conclusively determined. The diagnosis was lower EC because the tumor center was located in the lower esophageal region. Few reports have described esophageal cancer with NEC and AFP-producing adenocarcinoma components.^8,10,11)^ Furthermore, cases with features similar to the present case, which exhibit a variety of histologic features, have been rarely reported. The carcinogenetic pathway is not yet fully understood, although the differentiation of monoclonal multipotent stem cells into NEC or ENT components is a proposed mechanism.^12)^ In the present case, immunohistochemical staining revealed that the cells in the tumor center exhibited a poorly differentiated morphology and were positive for synaptophysin, chromogranin, glypican-3, and AFP, supporting this hypothesis. A pretreatment biopsy led to the suspicion of NEC, although the specimen was insufficient, a known limitation that can hinder definitive diagnosis due to sampling errors that fail to reveal all tumor components. The patient was treated with R2 radical esophagectomy, which, in addition to relieving the stenosis, provided adequate specimens for detailed analysis using various immunohistochemical stains. In this case, although no noncurative factors were detected on preoperative CT, the procedure resulted in a noncurative resection. It might have been better to repeat the CT scan immediately before the surgery. Despite radial surgery, the prognosis remained poor, and a detailed prognosis is still unknown.^4,13,14)^ Extensive metastases are often present at the time of diagnosis. Accordingly, chemotherapy is generally administered as the primary treatment for NEC with metastasis. The significance of surgery for esophageal NEC has yet to be fully established, as reflected in nationwide surveys by Sohda et al.^15)^. On the other hand, surgical specimens provide more extensive pathological information compared to the limited tissue obtained via biopsy; therefore, we believe this advantage does not necessarily preclude surgical treatment. However, specific treatment criteria for adjuvant therapy and chemotherapy in unresectable or recurrent lesions are lacking. In the present case, a regimen containing 5-FU, CDDP, and pembrolizumab was chosen, following the treatment guidelines for unresectable EC. The patient maintained CR with pembrolizumab monotherapy. **Figure 8** showed that all tumor markers were within the normal range at the 5th regimen, but the peaks for tumor markers AFP and SCC were different. The aggravation of underlying atopic dermatitis during treatment may have contributed to the elevation of SCC levels. A recent study reported increased programmed cell death ligand 1 expression in a high proportion of patients with NEC, whereas another study reported contradictory results.^16)^ Future studies are warranted to determine the utility of tumor microenvironment analysis in differentiating between NEC and ENT. Further investigation should also include clarifying drug susceptibility, regimen selection, and the significance of immune checkpoint inhibitors.

## CONCLUSIONS

We have experienced a case of AFP-producing hepatoid gastric adenocarcinoma with neuroendocrine differentiation. Though such a case has high biological malignancy, no specific treatment criteria for adjuvant therapy and chemotherapy in unresectable or recurrent lesions have been established as of now.

## DECLARATIONS

### Availability of data and materials

All the data in this article is included in the published article.
